# Comparative Study of Different Methods in Vibration-Based Terrain Classification for Wheeled Robots with Shock Absorbers

**DOI:** 10.3390/s19051137

**Published:** 2019-03-06

**Authors:** Mingliang Mei, Ji Chang, Yuling Li, Zerui Li, Xiaochuan Li, Wenjun Lv

**Affiliations:** 1 Department of Mechanical Engineering, Fujian Polytechnic of Information Technology, Fuzhou 350003, China; meimingliang@fjpit.edu.cn; 2 Department of Automation, University of Science and Technology of China, Hefei 230027, China; cjchange@mail.ustc.edu.cn (J.C.); lzerui@mail.ustc.edu.cn (Z.L.); 3 School of Computer Science and Information Engineering, Hefei University of Technology, Hefei 230601, China; lyl95@mail.hfut.edu.cn; 4 Faculty of Technology, De Montfort University, Leicester LE1 9BH, UK; xiaochuan.li@dmu.ac.uk

**Keywords:** autonomous robots, terrain classification, vibration, LSTM

## Abstract

Autonomous robots that operate in the field can enhance their security and efficiency by accurate terrain classification, which can be realized by means of robot-terrain interaction-generated vibration signals. In this paper, we explore the vibration-based terrain classification (VTC), in particular for a wheeled robot with shock absorbers. Because the vibration sensors are usually mounted on the main body of the robot, the vibration signals are dampened significantly, which results in the vibration signals collected on different terrains being more difficult to discriminate. Hence, the existing VTC methods applied to a robot with shock absorbers may degrade. The contributions are two-fold: (1) Several experiments are conducted to exhibit the performance of the existing feature-engineering and feature-learning classification methods; and (2) According to the long short-term memory (LSTM) network, we propose a one-dimensional convolutional LSTM (1DCL)-based VTC method to learn both spatial and temporal characteristics of the dampened vibration signals. The experiment results demonstrate that: (1) The feature-engineering methods, which are efficient in VTC of the robot without shock absorbers, are not so accurate in our project; meanwhile, the feature-learning methods are better choices; and (2) The 1DCL-based VTC method outperforms the conventional methods with an accuracy of 80.18%, which exceeds the second method (LSTM) by 8.23%.

## 1. Introduction

The current decade has witnessed an increasing application of autonomous vehicles and mobile robots in industrial transportation, ground reconnaissance, planetary exploration, etc. [1,2]. These autonomous robots performing outdoor tasks must face various terrain types. Hence, an accurate terrain classification is of significant importance to prevent the robots from non-geometric hazards which are mainly rendered by slippery and bumpy surfaces. For example, when a field robot is trying to traverse a slippery terrain, the terrain-dependent slippage coefficients can be estimated by means of terrain classification; consequently, excessive wheel sinkage can be avoided by slippage compensation algorithms, i.e., torque-based or speed-based traction control strategies [3]. In addition, terrain classification has been demonstrated to have great contributions in many other aspects, i.e., adaptive navigation, traction control, energy saving, gait control, etc. [4,5,6,7,8]. Therefore, the research on terrain classification have received great attention from the DARPA grand challenge and planetary exploration.

Robotic terrain classification refers to the process of a mobile robot identifying the current or forthcoming terrain, such as gravel, grass, etc., using its on-board sensors. The terrain classification methods can be categorized into two parts: the non-interactive and the interactive methods. The non-interactive methods are usually realized by optical sensors (e.g., digital camera, infrared camera and LiDAR) [9,10,11,12], which have been widely investigated. The non-interactive methods usually possess high classification accuracy, but they are demanding to environmental conditions (such as illumination), variations in appearance or cover (such as leaves). Alternatively, the interactive methods, which can be realized by means of acoustics [13,14,15], haptics [16,17,18] and vibration [19,20,21], are robust to changes in appearance and illumination, and have better computational efficiency. Therefore, the interactive methods have shown increasing potential in robotic environment perception. The acoustic terrain classification has been seldom studied due to a fatal issue of external environmental and internal mechanical noise [22]. The haptic terrain classification leverages ground reaction forces to recognize terrain, by means of tactile sensor arrays mounted on the robot-terrain contact area; therefore, they are more applicable to legged robots than wheeled robots [23]. Apart from the acoustic and haptic signal, vibration signal also provides sufficient information to discriminate different terrains based on mechanical properties [24]. In contrast to acoustic signal, the interference in accelerometer-gathered vibration signal is caused by gravitational acceleration, which is time-invariant and easy to eliminate. Additionally, vibration-based methods can be applied to both wheeled and legged robots compared with haptic ones. Considering the above advantages of vibration signal, we focus on VTC in this paper.

**Figure 1:**
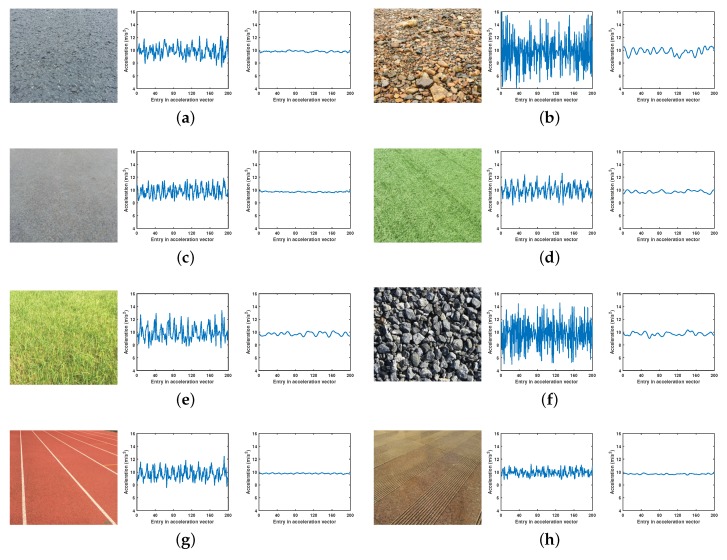
Examples of eight terrain types along with corresponding samples of undampened vibration signals (**left**) and dampened vibration signals (**right**). (**a**) Asphalt. (**b**) Cobble. (**c**) Concrete. (**d**) Grass 1 (artificial grassland). (**e**) Grass 2 (natural grassland). (**f**) Gravel. (**g**) Plastic. (**h**) Tile.

All the existing work concerning VTC concentrates on the robots with relatively hard tires and without shock absorbers, because the vibration signal could reflect the truth of ground surfaces. However, in real-world applications, to guarantee the stability and security of the robot itself, and protect the equipment or cargo on the robot, the robot is often equipped with elastic tires and shock absorbers. Hence, it is quite practical to study the VTC under the effect of shock absorbers. The difference between the vibration signals with and without shock absorbers can be seen in Figure 1. Obviously, the shock absorbers result in a significant dampening of vibration magnitude, especially the high frequency component, so the dampened vibration signal cannot reflect the fine surface characteristics. This means the terrains with small differences are difficult to discriminate from the dampened vibration signal. To the best of our knowledge, VTC problem under shock absorbers has not been investigated. Therefore, whether the existing relevant methods are applicable to dampened vibration signals should be verified. If not, more effective methods should be developed.

In this paper, we explore the VTC, in particular for wheeled robots with shock absorbers. Because the vibration sensors are usually mounted on the main body of the robot, the vibration magnitude are dampened significantly, which results in the vibration signals collected on different terrains being more difficult to discriminate. Hence, the existing VTC methods applied to a robot with shock absorbers degrades. The contributions are twofold:

1. A large body of comparative experiments are conducted to demonstrate the performance of the existing feature-engineering and feature-learning classification methods.
2. According to the long short-term memory (LSTM) network, we propose a one-dimensional convolutional LSTM (1DCL)-based VTC method to learn both spatial and temporal characteristics of the dampened vibration signals.

The real-world experiment on eight different terrains demonstrates that:

1. In feature-engineering approaches, the combination of power spectral density (PSD) and random forest (RF) achieves the highest accuracy of 69.88%.
2. The conventional feature-learning approaches outperforms the feature-engineering ones slightly by about 2%, though they are computationally intensive.
3. The proposed 1DCL-based VTC method outperforms the conventional methods in an accuracy of 80.18%, which exceeds the second method (LSTM) by 8.23%.

The remainder of this paper is organized as follows. Section 2 presents related work. In Section 3, we describe the framework of this paper and summarize the feature-engineering approaches. In Section 4, we give a brief introduction of feature-learning approaches and describe our approach in detail. The dataset, evaluation metrics and experiment results are then presented in Section 5. Finally, in Section 6, we draw conclusions and give proposals for future work.

## 2. Related Work

A large body of studies about non-interactive methods using exteroceptive modalities have been done. Image-based methods usually use digital cameras or infrared cameras. Digital image-based methods mainly extract visual features from terrain images using local binary patterns (LBP), bag of visual words (BOVW) or speeded up robust features (SURF) [10,11,25,26]. Recently, convolutional neural networks (CNNs) have attracted more attention due to their excellent feature extracting capability, and thus applying to near-range and far-range terrain classification and planetary surface soil property analysis [27,28]. Digital cameras may not perform well in the dark. In contrast, infrared cameras which usually cooperate with laser or ladar can work at night [29]. However, infrared image-based or ladar-based methods focus on segmenting the ground surface from different obstacles [30,31,32], rather than classifying the terrain type.

Interactive methods using proprioceptive modalities has not been explored in the same depth as non-interactive methods. The existing interactive methods are usually realized by acoustics, haptics, and vibration. Acoustic terrain classification is motivated by vehicle-terrain interaction sounds which is produced when the robotic locomotion mechanism (e.g., legs, wheels or tracks) is pressing the ground. This idea is first proposed by [22]. In their work, the acoustic features are extracted by surveying acoustic methods from other domains and their efficacy is verified in terrain classification. Adopting feature extraction methods from speech processing literature, a novel feature vector composed of zero crossing rate, spectral band energies and their vector time derivatives is proposed [13]. In [15], a deep spatiotemporal model is designed for learning complex dynamics in audio signals which replaces manually handcrafted features. Their model yields an average classification accuracy of 97.36% on the offline experiment. The acoustic terrain classification has a fatal issue of external environmental and internal mechanical noise, so it is impractical in noisy outdoor environment. One alternative modality, haptics, is most investigated on legged robots. Haptic feedback is obtained by force sensors or capacitive tactile sensors mounted on robotic legs [16,23]. According to the sensory information and the perceived terrain type, legged robots can perform gaits and speed adjustment for more effective motion. More work can be found in the literature [17,18].

The most popular proprioceptive modality in interactive method is vibration. Vibration signals are easily collected in contrast to haptic signals, since the classifier can perform well even with a consumer-grade accelerometer The early work that involves the VTC uses power spectrum as the feature [33]. In later work, researchers begin to develop more simple and compact features in the time domain instead of a more than 100-dimension frequency-domain feature [34]. An 8-dimensional feature vector composed of zero crossing rate, mean, standard deviation, etc., is then developed. This method costs less computing time than the spectral method; however, in some applications, these compact features are not sufficient to cover all the characteristics of the raw data. Support Vector Machine (SVM) has been demonstrated to be the best performing classifier using time- and frequency-domain features [21,34]. In [20], Dynamic Cortex Memory (DCM) which is an extension of LSTM is addressed for VTC without any explicit feature computation. Their experiments on 14 terrain types achieve an overall accuracy of approximately 85%, which is the state-of-the-art accuracy for 14-class terrain classification. Moreover, they implement just-in-time computation for their networks. Apart from terrain classification, vibration is also frequently used in damage detection field [35,36,37]. In their work, some use time-domain features with a specific goal, such as kurtosis, crest factor and Root Mean Square (RMS), and others use Discrete Fourier Transform (DFT) to obtain frequency-domain features. These literatures involved feature extraction methods and classification algorithms will appear in the comparative study.

Multi-modality methods combine several modalities based on their complementary characteristics, thus possessing a higher accuracy and robustness against environmental interference [38,39]. In [40], three-axis acceleration, roll-pitch-yaw (RPY), angular rates, and cloud point images are combined by using bagging algorithm. The results achieve a similar level of accuracy as SVM and visual sensor data, with less computation. In [41], five data sources including four vibration sources and one acoustic source are collected by their tracked robot for fusing predictions. A two-stage feature selection method that combines Relief and mRMR algorithms is developed to obtain optimal feature subsets. In addition, four different classifiers are combined for classification task.

In the following comparative study, we refer to some of the feature extraction methods and classification methods of the aforementioned literature.

## 3. Feature-Engineering Approaches

**Figure 2:**
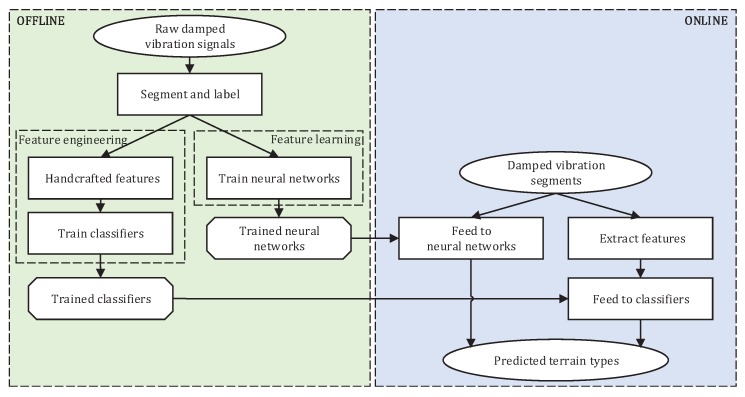
Illustration of dampened vibration-based terrain classification.

The dampened VTC is illustrated in Figure 2. The entire process is divided into the offline training and the online classification. In the offline process, the raw dampened vibration signals are used to train the classifiers; in the online process, the trained classifiers are used to predict the testing dampened vibration segments.

In this section, we introduce the feature engineering approaches.

### 3.1. Vibration Feature Extraction

We collected vibration data by an accelerometer mounted perpendicularly on the main body of a wheeled mobile robot with shock absorbers. In our experiment, we recover the vibration signal from the accelerometer readings by subtracting the gravitational acceleration, and then split the vibration signal into short segments. Each segment contains 50% overlap of successive segments. In Section 5, we evaluate different segment sizes that affect the classification accuracy.

Now we are in the position to extract features from each segment. Feature extraction is performed in the time domain and in the frequency domain. Time-domain vibration feature extraction method aims to obtain a simple and compact representation of vibration signal. In VTC, mean, standard deviation, norm, autocorrelation, RMS, maximum and minimum are usually selected as statistical features [34,42]. In vibration-based damage detection, RMS, skewness and kurtosis are used since they have been proved to be useful for bearing fault detection [35,43]. Acoustic terrain classification is similar to the vibration-based one, so we refer to its features such as zero crossing rate and short time energy [13,14,22]. Here, we summarize the most common time-domain features.

Consider the training sample set $D=\{v_{1},v_{2},\ldots ,v_{m}\}$, where *m* is the number of samples. For each sample $v_{j}=\left(v_{j,1},v_{j,2},\ldots ,v_{j,n}\right)\in D$, where *n* is the number of acceleration values in a segment, the time-domain features are the followings. Please note that we will omit subscript *j* later for brief. That is to say, symbol $v_{i}$ means the *i*th value of sample v.

(1) Zero crossing rate (ZCR). The ZCR counts the number of times that the signal crosses the zero axis, which is an approximate estimation of the frequency of v.
(2) Mean. The mean expresses the average roughness of ground surface.
(3) Standard deviation. Intuitively, the standard deviation is greater with a rougher ground surface.
(4) Norm. Usually the $\ell_{2}$-norm is used which reflects the energy of v.
(5) Autocorrelation. The autocorrelation *r* is a measure of the non-randomness as defined by
  (1) $$r=\frac{1}{n\sigma^{2}}\left(\sum_{i=1}^{n-1}\left(v_{i}-\mu \right)\left(v_{i+1}-\mu \right)\right),$$
  where *r* gets larger with the dependence of successive vibration values.
(6) Maximum. Find the maximum of the degree of bump.
(7) Minimum. Find the minimum of the degree of bump.
(8) Skewness. The skewness Sk describes the asymmetry of the distribution about its mean, calculated as
  (2) $$Sk=\frac{\left[\frac{1}{n}\sum_{i=1}^{n}{\left(v_{i}-\mu \right)}^{3}\right]}{\sigma^{3}}.$$
(9) Excess kurtosis. The excess kurtosis EK reflects the degree of deviation from the Gaussian distribution as defined by
  (3) $$EK=\frac{1}{n}\frac{\left[\frac{1}{n}\sum_{i=1}^{n}{\left(v_{i}-\mu \right)}^{4}\right]}{\sigma^{4}}-3.$$

Frequency-domain representation is widely used in signal processing field as it simplifies the mathematical analysis and helps analyze the components of signals. The most common tool for frequency-domain transformation is Fast Fourier Transform (FFT) that is an extensive application in both VTC [44,45] and acoustics-based terrain classification [15,22]. PSD that describes how power of a signal is distributed over frequency is also used for processing vibration signals [33,42]. In this paper, we perform both FFT and PSD to convert vibration signals from time domain into frequency domain. FFT is an efficient computing algorithm for DFT. Given a sequence x(i) of length *n*, the N-point DFT of x(i) is defined as

(4) $$X\left(k\right)=D{\left\{x\left(i\right)\right\}}_{N}=\sum_{i=0}^{N-1}x\left(i\right)e^{-j\frac{2\pi }{N}ki},k=0,1,\ldots ,N-1.$$

Usually N≥n and x(i) is zero-padded which means the terms from $a_{n+1}$ to $a_{N}$ are padded with zero. Typically, *N* is specified as a power of 2. Hence, the training vectors of different lengths in our experiments implement the corresponding points FFT. PSD is defined as the Fourier transform of the autocorrelation function. For limited discrete values, the PSD of X(i) denoted by $S_{X}\left(\omega \right)$ is estimated by

(5) $$S_{X}\left(\omega \right)=D\left\{R_{X}\left(k\right)\right\}=\sum_{k=-N}^{N}R_{X}\left(k\right)e^{-j\omega k}$$

where $R_{X}\left(k\right)$ denotes the autocorrelation function of X(t). A log scaling of the PSD is implemented in this study to reduce the dominating effect of high-magnitude frequency components.

### 3.2. Classifiers

Various classifiers have been used for VTC and other fields. Among them, SVM is used the most widely [21,34,39,45]. Other common classifiers such as k-nearest neighbor (kNN), decision tree (DT), Naïve Bayes (NB) and extreme learning machine (ELM) are also applied [40,44,46]. Additionally, ensemble learning, e.g., RF and AdaBoost, is a research hotspot in machine learning for its superior generalization performance [10,47]. In our experiment, we adopt seven classifiers: SVM [48], ELM [49], kNN (Classic kNN determines the class by the majority vote, which suffers from sample imbalance. Therefore, instead of using classic kNN, we employ a centroid displacement-based k-nearest neighbors algorithm [50] which has been proved to be adaptive to noise and class distributions) [50], NB [51], DT [52], RF [53] and AdaBoost [54]. KNN is a benchmark method in our study.

## 4. Feature-Learning Approaches

The classification performance of feature-engineering approaches relies on handcrafted feature extraction heavily. In recent years, there has been a considerable effort on the development of end-to-end learning methods [55]. Instead of manually extracting characteristic features, end-to-end learning method can learn the discriminative feature representation directly from raw data. The latter approach does not require too much prior knowledge of the problem or human expertise, and is advantageous in tasks where some high-level, abstract features from raw data are almost impossible to be developed manually. Usually, end-to-end learning method like deep neural network suffers from computationally intensive training process. However, once the network is trained, it can be directly assembled to the mobile robots, thus not computationally intensive.

In this section, we first introduce two widely used end-to-end feature-learning methods: LSTM and CNN. Next, we propose a novel neural network architecture which is called 1DCL.

### 4.1. Overview of CNN and LSTM

#### 4.1.1. Convolutional Neural Network

A typical convolutional neural network consists of alternating convolution and subsampling layers after the input layer and a generic multilayer network (fully connected layers) at the last stage of the architecture. At a convolution layer, the input feature maps from the previous layer are convolved with kernels which should be learned, and then the convolved results are put through the activation function to form the output feature map. After feature extraction in the convolution layers, the output feature map is transferred to subsampling layers (also named pooling layers) for feature selection and information filtering. The last few layers are fully connected layers that are identical to a multilayer perceptron (MLP) for estimating the decision (classification) vector. In CNNs, each convolutional neuron is only connected to some of the nodes in the previous layer called receptive field, which is a main characteristic of CNNs named sparse connection. Another characteristic of CNNs is parameter sharing that all the units in a feature map share the same parameters. These parameters constituted matrix refers to as kernel.

#### 4.1.2. Long Short-Term Memory

Recurrent neural networks (RNNs) can process and predict sequence data due to their recurrent structure [20]. However, traditional RNNs are unable to solve long-term dependencies problem and often suffer from vanishing and exploding gradient problems during training. The LSTM architecture [56] can overcome vanishing gradient problem effectively and enables exploitation of long-term temporal dynamics of a sequence.

The LSTM contains special units called memory blocks (mentioned as LSTM cells in the following) in the recurrent hidden layer. The LSTM cells contain a memory cell $c_{t}$ used to store the temporal state of the network and three gates (at each time step *t*) called forget gate $f_{t}$, input gate $t_{t}$ and output gate $o_{t}$, respectively. The forget gate controls the forgotten states of the previous LSTM cell, the input gate controls the input activations into the LSTM cell, and the output gate controls the output of cell activations. The LSTM transition equations are the following:

(6) $$\begin{array}{cc} i_{t} & =\sigma (W_{ix}x_{t}+W_{ih}h_{t-1}+b_{i})\\ f_{t} & =\sigma (W_{fx}x_{t}+W_{fh}h_{t-1}+b_{f})\\ o_{t} & =\sigma (W_{ox}x_{t}+W_{oh}h_{t-1}+b_{o})\\ u_{t} & =\tanh(W_{ux}x_{t}+W_{uh}h_{t-1}+b_{u})\\ c_{t} & =i_{t}⊙u_{t}+f_{t}⊙c_{t-1}\\ h_{t} & =o_{t}⊙\tanh\left(c_{t}\right) \end{array}$$

where $x_{t}$ is the current input vector, $h_{t}$ is the current cell output activation vector, $u_{t}$ is the candidate value, *W* and *b* are weight vectors and bias vectors. σ and tanh denote the logistic sigmoid function and the hyperbolic tangent function, respectively. ⊙ denotes element-wise multiplication.

### 4.2. Proposed 1DCL

To our knowledge, the CNN-based and LSTM-based VTC have rarely been investigated. Many existing CNN and LSTM models are often general-purpose, thus not suitable for our application. In our study, the dampened vibration signals increase the difficulty of terrain classification, so we design a dedicated neural network model called 1DCL by modifying and integrating CNN and LSTM. Such a neural network model can learn both spatial and temporal characteristics of the raw vibration signals.

**Figure 3:**
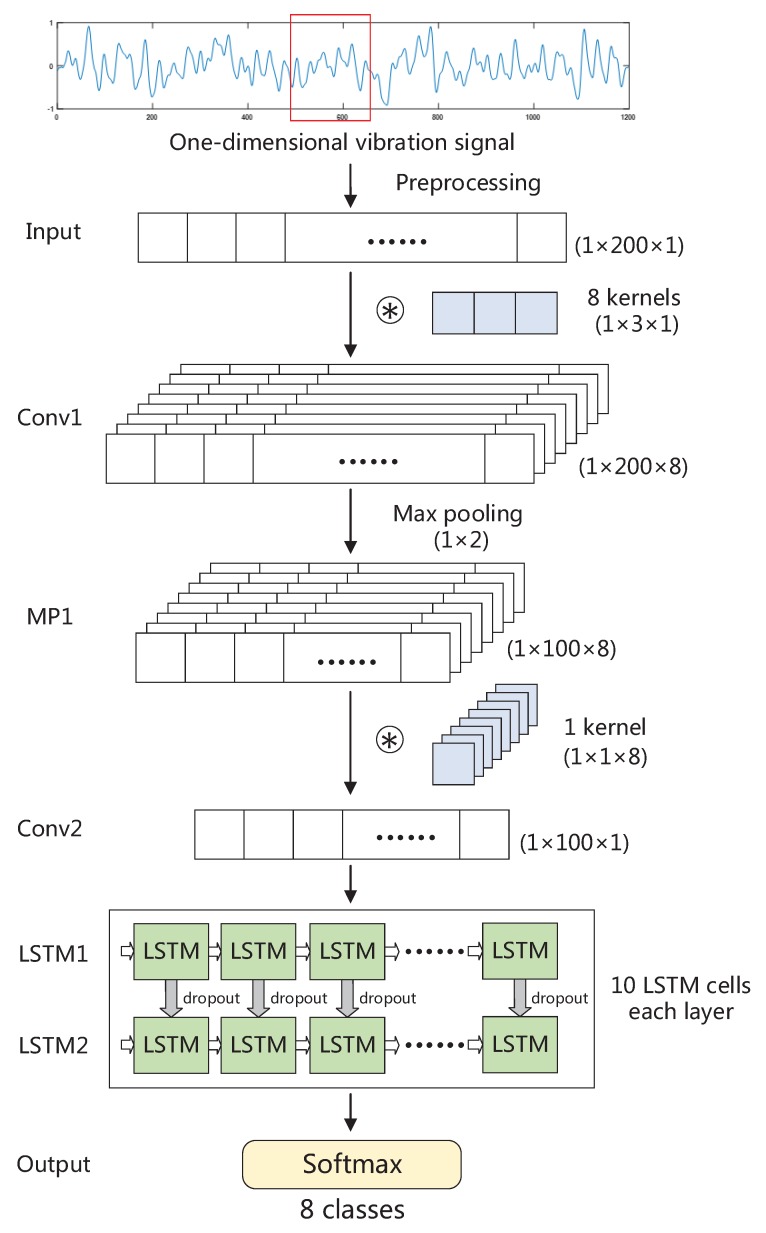
The overall structure of 1DCL. The marker ⊛ denotes convolution operation.

The proposed 1DCL shown in Figure 3 consists of a 1D convolutional layer connecting with a max pooling layer, a 1×1 convolutional layer and a two-layer LSTM and a SoftMax layer. Before fed into the 1DCL, the input vibration signals are preprocessed. Just as with previous operations, the raw vibration signals are split into short segments with 50% overlap of successive segments, and each segment contains 200 acceleration values. In addition, we normalized the segments into zero mean and unit standard deviation, which is a common procedure for neural networks that usually leads to the best classification performance.

We employ a 1D convolutional layer in the 1DCL to learn preliminary spatial features. Structural differences between the traditional 2D and the proposed 1D CNN are visible, that is, the usage of 1-D arrays replace 2-D matrices for both kernels and feature maps. In the 1DCL, convolution kernel of the 1D convolutional layer is of size 3. Convolutional stride is fixed as 1. A max pooling layer with a kernel of size 2 follows the 1D convolutional layer. Max pooling layer provides some invariance and reduces parameters, thereby being able to avoid overfitting. The 1×1 convolution which is first proposed in [57] can change the number of the channels of feature maps without changing the length and width of feature maps. Here we employ a 1×1 convolutional layer after the convolutional and pooling layer, to reduce the number of channels of feature map into 1 (i.e., dimension of 1×100×1). In the two convolutional layers, we applied rectified linear unit (ReLU) as the nonlinear activation function. The ReLU has been proved to solve the vanishing gradient problem. Besides, it expedites the convergence of the training procedure compared with sigmoid and tanh activation function [58].

Rather than constructing multiple convolutional layers to learn deep spatial features, we only build the above two convolutional layers because we pay more attention to learn temporal dynamics from the vibration sequence. As shown in Figure 3, a two-layer LSTM is stacked after the 1×1 convolutional layer. At each time step of the feature map, the outputs of the previous LSTM cell ($c_{t}$ and $h_{t}$ in Equation (6)) are used as the inputs of the LSTM cell at next time step. The output activation vector of the LSTM cell $h_{t}$ in the first layer is used as the input of the LSTM cell in the second layer. For each layer, there are ten cells. Dropout regularization is used between the two LSTM layers to avoid overfitting with dropout probability of 0.5. Finally, a SoftMax layer is used to output the classification results.

As for the hyperparameters, we adopt the Spearmint Bayesian optimization library [59] to tune the hyperparameters in the artificial neural network, and grid search combined with 5-fold cross-validation to tune the hyperparameters in the feature-engineering approaches (e.g., SVM, ELM).

## 5. Experiment and Results Analysis

To compare different classification methods applied in terrain classification, we conduct the experiment with a four-wheeled mobile robot on 8 different terrains.

### 5.1. Experiment Setup

**Figure 4:**
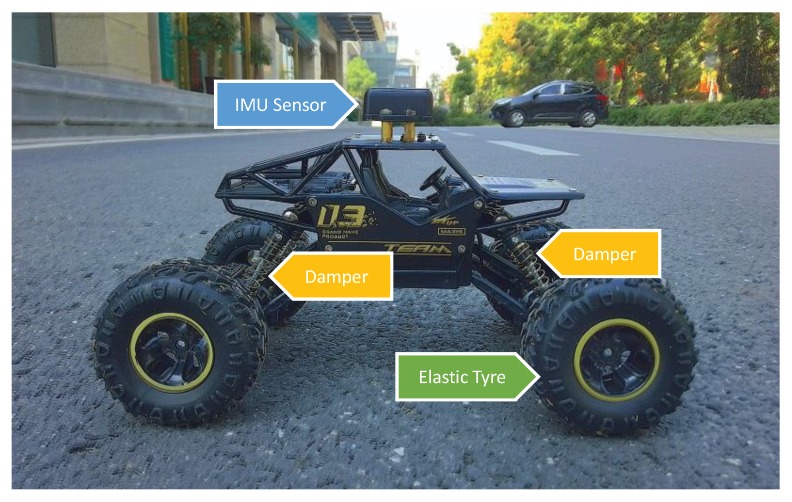
The experimental robot.

The experimental robot is shown in Figure 4. The robot is equipped with 4 dampers and 4 elastic tires, which constitute the robotic shock absorber. An IMU sensor is mounted on the robot roof to perceive the damped vibration.

We collect vibration data by controlling the four-wheeled mobile robot to wander on eight terrain types which are different in rigidity, roughness, and flatness. Some of them are artificial terrains (e.g., asphalt, artificial grassland), while some are natural ones (e.g., cobble, natural grassland). In our experiment, we recover the vibration signal from the accelerometer readings by subtracting the gravitational acceleration, and then split the vibration signal into short segments. The length of each segment is set as 200 points initially, and each segment contains 50% overlap of successive segments; consequently, the dataset contains 11,224 samples in total (Asphalt: 1241; Cobble: 1434; Concrete: 1322; Artificial grassland: 1434; Natural grassland: 1562; Gravel: 1222; Plastic: 1624; Tile: 1385). Figure 1 shows the photos of the eight terrain types, along with the corresponding samples of undampened vibration signals and dampened vibration signals. Obviously, the shock absorbers result in a significant dampening of vibration magnitude, especially the high frequency component, so the dampened vibration signal cannot reflect the fine surface characteristics. The experimental robot traverses the eight terrains at a speed varying between 0.7 and 1.1 m/s, and in different motion mode (e.g., circular and linear motion) to prevent the classifiers from overfitting a certain motion.

### 5.2. Experiment Results and Analysis

In this section, we first study the performance of different classifiers in combination with handcrafted vibration features, i.e., feature-engineering approaches. Each pair of handcrafted feature and classifier is evaluated to find out the best combination. We also analyze the performance of 1DCL and compare it with feature-engineering approaches and other feature-learning approaches. Finally, experiments on different segment lengths are done.

**Figure 5:**
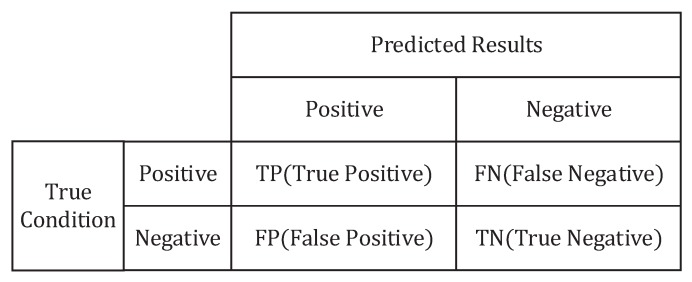
Confusion matrix of binary classification. The confusion matrix of multi-class classification can be reduced to several confusion matrices of binary classification.

The 10-fold cross-validation is used to evaluate the classification performance by three parameters, namely, accuracy, True Positive Rate (TPR) and F1-score. The accuracy Ac, which indicates the overall capability of the classifier to identify different terrains correctly, is given as

(7) $$Ac=\frac{N_{c}}{N_{t}}$$

where $N_{c},\;N_{t}$ denote the number of correctly classified samples and the total number of the testing set, respectively. We focus on not only the overall capability of a classifier, but also the discriminability of each class which are evaluated by the indices of precision and recall. The precision Pr evaluates the correct rate predicted by the classifier for a certain class, and the recall Rc evaluates the proportion of correctly classified samples in class of interest, as follows

(8a) $$Pr=\frac{TP}{TP+FP},$$

(8b) $$Rc=\frac{TP}{TP+FN},$$

where the definitions of TP, FP and FN are illustrated in Figure 5. The TPR has the same meaning as the recall, so it is also calculated by Equation (8b). The F1-score takes both precision and recall into account.

We have

(9) $$F1=\frac{2\times Pr\times Rc}{Pr+Rc}.$$

The calculation of Pr, Rc, and F1 shown above are used in binary classification. We can simply apply the macro-averaging technology [60], i.e., averaging Pr, Rc, and F1 of each class, to extend these metrics in multi-class classification.

#### 5.2.1. Analysis of Performance of Feature-Engineering Approaches

Table 1 shows the accuracy and F1-score of seven different classifiers using time-domain and freq-domain (including FFT-based and PSD-based) feature extraction methods. In general, random forest classifier using PSD-based features (PSD-RF for short) achieves the best performance of 69.88% accuracy and 0.6897 F1-score. With respect to the features, PSD is the best description of the characteristics of vibration signals overall, followed by FFT. Time-domain statistical features seem not to express the essential characteristics very well. One possible reason for the difference between freq-domain and time-domain features is that the complexity and irregularity of undampened vibration signals in time domain make it difficult to describe the most distinguishing characteristics. However, when transformed to frequency domain, complex vibration signals are decomposed into several single harmonic components, which facilitates the description of the characteristics. As for the classifiers, the performance of AdaBoost, ELM, RF, and SVM are similar, outperforming the other three classifiers significantly. SVM was found to be the most accurate method for vibration-based classification in [21,34], while ELM have been proved to have good generalization capability [49]. Naïve Bayes and DT have weak learning ability for our dampened vibration data. Their performance are even worse than the benchmark kNN in terms of time-domain features and PSD-based features. Results can be improved through some ensemble approaches, e.g., RF and AdaBoost. As shown in Table 1, RF and AdaBoost based on weak learner of DTs improve the prediction accuracy by over 7.5%.

**Figure 6:**
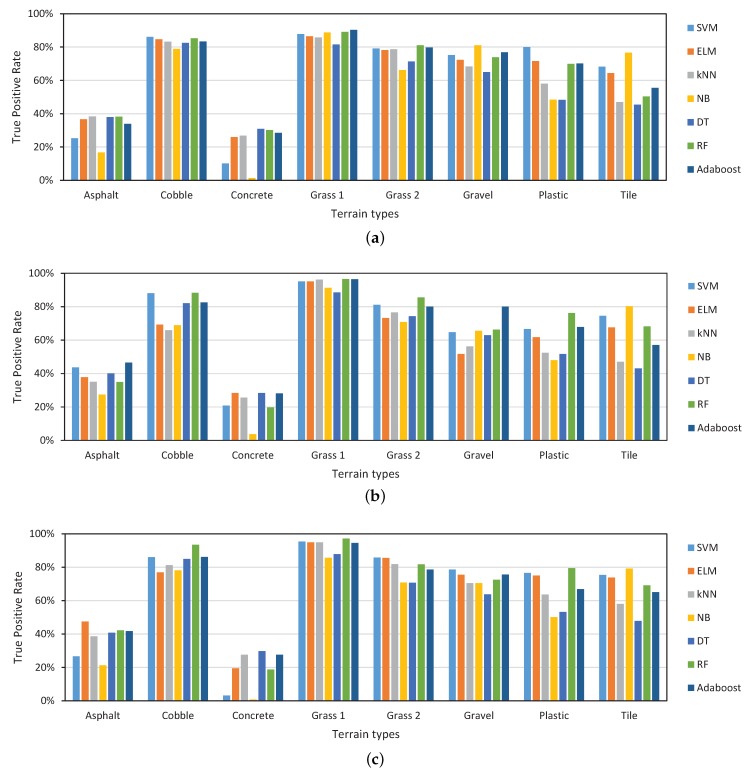
True positive rate for 8 terrain types with different classifiers using (**a**) Time-domain features, (**b**) FFT-based features, (**c**) PSD-based features.

**Figure 7:**
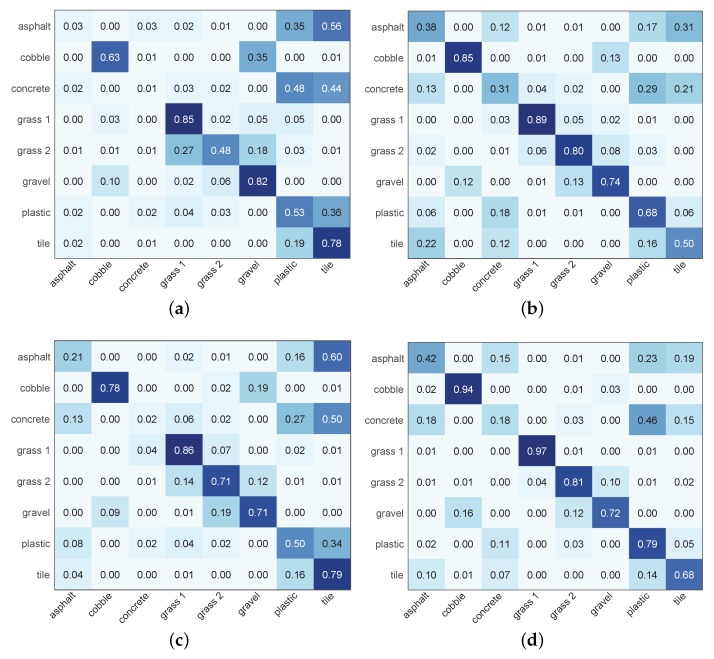
Normalized confusion matrices of (**a**) T-NB, (**b**) T-RF, (**c**) PSD-NB, (**d**) PSD-RF.

Considering the ability to distinguish each terrain type, as illustrated in Figure 6, the TPR for grass 1 of each classifier is the highest overall, which demonstrates that grass 1 is the easiest to identify. For cobble, grass 2, gravel, plastic, and tile, the TPR is also relatively high. However, for asphalt and concrete, the TPR is always low. To further investigate the terrain types to which asphalt and concrete are misclassified, Figure 7 presents the confusion matrices of RF and NB using time-domain features and PSD-based features. For the space limit, we only exhibit the above 4 representative combinations. Asphalt and concrete are most likely to be misclassified to plastic and tile. Even in the best combination PSD-RF shown in Figure 7d, asphalt is misclassified to plastic for 23%, tile for 19% and concrete for 15% of the test cases, and concrete is misclassified to plastic for 46%, tile for 15% and asphalt for 18% of the test cases.

#### 5.2.2. Analysis of Performance of Feature-Learning Approaches

Now we are in the position to evaluate the performance of feature-learning approaches. The experiments are conducted on a system with a NVIDIA GeForce GTX 1080 Ti GPU and an Intel i7-7700K processor. We perform the experiments using different neural network model configurations to gain insight on the effect of learning spatiotemporal relationships. We consider the following five model configurations:

- 1D-CNN. A one-dimensional convolutional neural network with alternating convolutional and pooling layers learns spatial features of a segment, and is followed by two fully connected layers and a SoftMax layer.
- LSTM. A single layer LSTM model directly handles 200 points length segments with 10 LSTM cells in series, which helps to learn temporal dynamics.
- 1DCL. The proposed model is a spatiotemporal architecture.
- 1DCL-FC. A variant of our 1DCL that we use a fully connected layer to replace the 1×1 convolutional layer.
- 1DCL-3Conv. Another variant of our 1DCL model that we consider increasing the number of convolutional layers to learn better spatial features. Here, we build two normal convolutional layers and a 1×1 convolutional layer for a total of 3 convolutional layers.

Results from the comparison are shown in Table 2. The proposed 1DCL outperforms all the other models by achieving an accuracy of 80.18%, the best results of feature-engineering approaches by 10.30%, and the second method (LSTM) by 8.23%. The results demonstrate that the spatiotemporal architecture we designed indeed learns essential characteristics and complex dynamics in proprioceptive signals. The CNN and LSTM achieve accuracies of 70.17% and 71.95%, which are both better than the best results of feature-engineering approaches. Hence, end-to-end learning methods can be employed to improve the performance and replace manually selecting features process in terrain classification field, which however, is rarely investigated currently.

The two variants of our 1DCL, 1DCL-FC, and 1DCL-3Conv, do not perform as well as the 1DCL seen from Table 2. Increasing the number of convolutional layers does learn better spatial features, yet increases the complexity of the model. Hence, the model is more likely to overfit. In contrast to 1×1 convolution, fully connected layer has more parameters to train. Moreover, 1×1 convolution keeps the structure of feature maps, in other words, each position of the output feature map of the 1×1 convolutional layer corresponds to the same position of input feature maps. However, if replaced by a fully connected layer, the structure of feature maps is restructured, which may be the main reason for the relatively low accuracy of the 1DCL-FC.

#### 5.2.3. Comparison of Classification Time

Since wheeled robot with a terrain-dependent control system works in outdoor environments, terrain classification must be implemented in real time to ensure the safety of the robot and make the robot adapt its driving style to the current terrain. As depicted in Figure 2, training process is done offline while prediction is done online, thus the time for classifying a test vector is more important. Training time is considered only in some situations when the trained model should be updated or retrained. Table 3 compares the computation times of different approaches. It is well known that the deep neural networks need a time-consuming training process. The training time of SVM is the highest in the feature-engineering approaches because the grid search to determine the optimal parameters is also very time-consuming. These computationally intensive training process can be done offline. As for online testing, using NB and DT, it takes only a few milliseconds. ELM, kNN, RF, and the three neural networks take no more than 40 milliseconds other than FFT-kNN. However, the test time of SVM and AdaBoost is significantly higher. On the other hand, in view of features, FFT and PSD take more time than time-domain features whatever training or testing.

#### 5.2.4. Comparison of Varying Segment Length

At the end of this section, we perform experiments using varying vibration signal segment length on two representative approaches: FFT-SVM and the proposed 1DCL. Please note that splitting raw vibration signal into segments with different lengths will result in different number of instances. Therefore, we randomly select the same number of instances for each experiment to ensure the comparability of the experiment results, which may correspondingly cause a slight drop for the accuracy due to the reduction in training samples.

**Figure 8:**
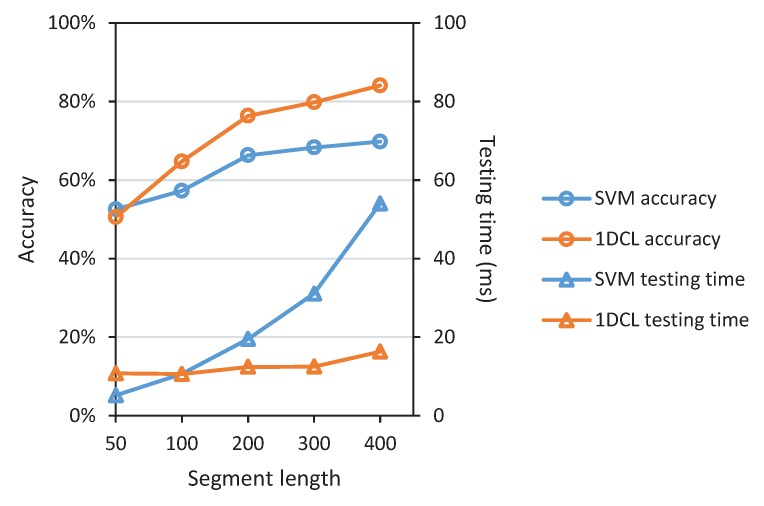
Accuracy and testing time of different approaches at varying vibration signal segment length. We take two representative approaches as examples: FFT-SVM and 1DCL.

Evaluating the segment sizes presents a resolution-efficiency trade-off. That is, a longer segment length contains more information of ground surface thus yielding increased accuracy requires more execution time, while a shorter segment length with faster real-time response leads to worse classification results. For applications of terrain classification, fast response and execution rates are essential for traversability evaluating. Figure 8 shows the classification accuracy at varying segment length and the corresponding testing time. It is observed that increasing the segment length does increase the accuracy. This growth slows down after the segment length exceeds 200. On the contrary, the testing time of SVM increases greatly with the segment length increasing. Interestingly, the testing time of the proposed 1DCL model does not seem to have a significant response to the varying segment length. Considering the segment length to classification time trade-off, we choose the segment length of 200 (i.e., 1 s) for the in-depth performance evaluation experiments as described above.

## 6. Conclusions

In this paper, we conducted an extensive comparative study of VTC with shock absorbers. We presented a large body of experiments on feature-engineering approaches including 21 combinations of 3 kinds of features and 7 different classifiers, and feature-learning approaches including 3 neural networks. Referring to this study, one can find the most appropriate classification method according to their demands. If there are no limits on computational complexity, the proposed 1DCL could be the best solution. In future work, we will enhance the proposed 1DCL to implement real-time computation, which is indispensable for outdoor mobile robots, referring to literature [20]. Additionally, we will employ semi-supervised learning to solve the issues of the lack of labels in terrain classification.

## Figures and Tables

**Table 1:** Performance of Seven Different Classifiers Using Time-Domain and Freq-Domain Feature Extraction Methods.

Features	Metrics	Classifiers						
		SVM	ELM	kNN	NB	DT	RF	Adaboost
Time-domain	Accuracy	65.30%	65.95%	61.53%	57.96%	58.39%	65.89%	**66.07%**
	F1-score	0.6438	**0.6497**	0.6065	0.5617	0.5803	0.6475	0.6465
FFT-based	Accuracy	67.99%	62.96%	57.75%	57.67%	59.62%	68.07%	**68.12%**
	F1-score	0.6674	0.6068	0.5695	0.5612	0.5898	0.6679	**0.6717**
PSD-based	Accuracy	67.52%	69.84%	65.69%	58.37%	60.26%	**69.88%**	67.91%
	F1-score	0.6622	0.6853	0.6454	0.5595	0.5999	**0.6897**	0.6699

**Table 2:** Performance of Feature-Learning Approaches.

Metrics	Networks				
	CNN	LSTM	1DCL	1DCL-FC	1DCL-3Conv
Accuracy	70.17%	71.95%	**80.18%**	67.94%	72.93%
F1-score	0.6893	0.7030	**0.7878**	0.6622	0.7159

**Table 3:** Running Time of Feature-Engineering and Feature-Learning Approaches.

Classifiers	Training Time (s)			Testing Time (ms)		
	T	FFT	PSD	T	FFT	PSD
SVM	15.43	188.9	90.20	18.78	235.2	115.4
ELM	0.5709	1.401	0.5674	19.93	27.20	21.72
kNN	0.01219	0.01956	0.01423	8.298	69.40	36.63
NB	$8.120\times 10^{-3}$	0.03499	0.01702	1.225	4.144	1.779
DT	0.01089	0.1596	0.08480	0.1409	0.4181	0.3472
RF	1.5371	22.80	11.81	16.35	10.71	9.431
Adaboost	6.911	173.1	82.26	81.54	96.39	82.07
CNN	251.8			7.110		
LSTM	746.5			13.18		
1DCL	1295			15.22		
